# Social Course Patterns of Urban Dwellers with Tuberculosis under Fragile Living Conditions in Tokyo, Japan

**DOI:** 10.2188/jea.16.167

**Published:** 2006-07-13

**Authors:** Masashi Kizuki, Takehito Takano, Keiko Nakamura, Yoshiharu Fukuda, Masafumi Watanabe, Tomoko Inose, Kaoruko Seino, Yoshiko Kawabe

**Affiliations:** 1 Health Promotion, Department of International Health Development, Graduate School of Tokyo Medical and Dental University.; 2 International Health and Medicine, Department of International Health Development, Graduate School of Tokyo Medical and Dental University.; 3 National Hospital Organization Tokyo National Hospital.

**Keywords:** Tuberculosis, fragile living, Alcohol Drinking, treatment interruption, Drug Resistance

## Abstract

**BACKGROUND:**

People under fragile-living conditions show a high rate of interruption of tuberculosis treatment. We examined the social courses of fragile-living urban dwellers with tuberculosis without customary and regular access to a conventional residence and investigated the factors associated with interruption of treatment.

**METHODS:**

One hundred and nineteen tuberculosis patients without customary and regular access to a conventional residence who were discharged from a hospital with the largest number of tuberculosis beds in Tokyo between January 1998 and October 2000 were followed up. The associations between demographic, social, and clinical characteristics and interruption of treatment were examined.

**RESULTS:**

The subjects (mean age, 51.2 years) were followed up for a median of 342 days. The percentage of cases of interruption of treatment during inpatient care among patients with alcohol problems (56%) was significantly higher than that among patients without such problems (11%). The proportion of cases of interruption of treatment during outpatient care among patients who were literally homeless before admission (40%) was significantly higher than that in others (5%), and that among those who used transient hostels after the initial inpatient treatment (55%) was significantly higher than that in others (4%). The prevalence of drug resistance was higher in cases with than without a history of tuberculosis treatment (P<0.05).

**CONCLUSIONS:**

Factors associated with interruption of tuberculosis treatment in patients under fragile-living conditions were identified. Interruption during inpatient care was significantly associated with alcohol problems, and interruption during outpatient care was significantly associated with the use of transient hostels.

Tuberculosis in urban dwellers without customary and regular access to a conventional dwelling or residence is a serious concern. People living under fragile conditions generally have a high risk of tuberculosis infection,^1^^-^^3^ and the affected individuals in such marginalized populations spread the disease in urban communities.^4^ The control of tuberculosis in people living under fragile conditions has become an urgent public health issue as a result of the increase in size of this population.

Adherence to the treatment regimens is essential for the control of tuberculosis, as it aids in early recovery from the disease, reduces the risk of disease reactivation, and prevents transmission of the disease within communities.^5^^,^^6^ However, people under fragile living conditions show a high rate of interruption of tuberculosis treatment.^5^^,^^7^ Thus, analysis of treatment interruption in tuberculosis patients under fragile-living conditions will increase our understanding of the factors related to a lack of adherence to treatment regimes in such marginalized populations.

There is a concern that people living in poor conditions first contact medical care in the late stage of the disease.^8^ In the case of tuberculosis infection, delay in seeking medical care implies an increased risk of spread of disease to other members of communities. The severity of tuberculosis in people living under fragile conditions is suggested by their high mortality, although previous studies have rarely revealed such information.

People under fragile living conditions are mobile. In general, follow-up of their treatment and outcome is difficult. In Tokyo, these people make use of various places for overnight stays, including the streets, bunkhouses for construction workers, transient hostels, and 24-hour public saunas.^9^ The use of complete information from hospitals and public health centers for individual patients allowed us to identify the treatment course in terms of use of inpatient and outpatient care and directly observed therapy, treatment completion, abandonment, and interruption of treatment by tuberculosis patients under fragile living conditions. We have referred to the treatment course as the ‘social course’ in tuberculosis patients under fragile living conditions, according to the concept of the social course of illness by Kleinman,^10^^,^^11^ which refers to the influence of aspects of the social environment on the course of recovery from illness.

The current lack of information on the social course of tuberculosis patients under fragile conditions has prevented the development of effective countermeasures to achieve successful treatment in such populations. There is an urgent need for studies on social factors influencing interruption of treatment in patients who lack a conventional residence, although there are various technical obstacles to obtaining comprehensive information on these patients.^12^ In-depth interview on the social conditions of patients on the basis of understanding the social characteristics of people under fragile living conditions allows the collection of such information.

This study was performed to determine the social course of tuberculosis patients under fragile living conditions and to investigate the demographic, social, and clinical factors associated with interruption of treatment and death during treatment in this population.

## METHODS

### Subjects and Setting

Subjects were patients of a hospital in Tokyo. The hospital has 220 beds for tuberculosis, the largest number allocated by any hospital in the Tokyo area. When people under fragile-living conditions are found or suspected to have tuberculosis, they are referred to hospitals with tuberculosis beds for further examination or treatment of tuberculosis. The hospital is a core hospital for treatment of tuberculosis in Tokyo, and it admits patients from in and around the Tokyo area.^12^ The standard treatment regimens include combinations of four drugs (isoniazid, rifampin, and pyrazinamide with ethambutol or streptomycin) or three drugs (isoniazid and rifampin with ethambutol or streptomycin).

The population eligible for this study were patients whose registration of residency was assigned to the address of the hospital of their admission, because of the lack of a conventional residence. Living conditions of these patients are poor, and they transient between living on the streets - literally homeless, transient hostels, temporary shelters, residence near their workplace, shabby apartments, and others.^13^ Therefore, in this study, we defined these patients as “patients under fragile-living conditions”.

Patients who met the criteria were consecutively selected from the discharge records between January 1998 and October 2000. Among 123 patients selected, four patients were found to be HIV-positive; data from the remaining 119 subjects, 103 of whom were discharged alive and 16 who died in hospital, were collected and used in this study.

### Data Collection

The data of the subjects were collected from their date of hospitalization, and followed during both their inpatient care and outpatient treatment. The hospital staff conducted face-to-face in-depth interviews with each of the 119 subjects and collected information on demographic and social characteristics with a predefined form. The staff have had training on interviewing patients with social problems including those with poor living conditions. Clinical characteristics were also evaluated and recorded by a physician. Demographic characteristics included sex and age on admission. Social characteristics included residence just prior to admission, residence immediately after discharge, primary occupation just prior to admission, existence of people with a close relationship who the patient expected to support him financially, and reason for access to medical services. Clinical characteristics included previous tuberculosis treatment, results of x-ray examination, results of sputum smears on admission, drug susceptibility of *Mycobacterium tuberculosis* isolate collected on admission against four drugs (isoniazid, rifampin, ethambutol, and streptomycin), and comorbidity of diabetes mellitus and alcohol problem in the hospital. The results of sputum smear were classified as ‘-’, 1+, 2+, and 3+, equivalent to the Gaffky scale of 0-1, 2-4, 5-8, and 9-10, respectively. Alcohol problem was the most frequently reported problem behavior, and a patient was defined to have an alcohol problem when they drank alcohol during their hospitalization and had trouble with other patients and the hospital staff.

To determine the social course of the subjects, we examined the reasons for discharge from the study hospital, and monitored the course of inpatient and outpatient treatment at medical and public health facilities for each patient after they left the hospital. Under the Tuberculosis Control Law, all cases of the disease must be reported to a local public health center by a physician within 2 days after diagnosis. The public health centers create a patient registration card and monitor each patient in their health jurisdiction. Then we identified the duration of treatment given to individual subjects as inpatient care and outpatient care, including directly observed therapy (DOT) in the community, and determined the final outcome. The follow-up process was terminated in April 2003.

The patients were categorized according to the reason for discharge from hospital: discharge after treatment completion (discharge after completion of the full course of treatment in the hospital based on the advice of physicians); transfer after treatment completion (transfer after completion of the full course of treatment in the hospital based on the advice of physicians); discharge for outpatient care (normal discharge from hospital followed by outpatient treatment); transfer (transfer to another hospital); voluntary discharge (leaving the hospital without permission or failure to return from a temporary discharge); forced discharge (unplanned early discharge because of poor behavior in the hospital, including violence); and death (defined as death due to any cause in the hospital before completion of treatment).

The patients were categorized into four groups according to the final outcome of tuberculosis treatment: treatment completion (defined as completion of planned tuberculosis treatment reported by physicians); abandonment (defined as a patients’ failure, by the end of at least 30 months of follow-up, to resume tuberculosis treatment after interruption of treatment); death (defined as death due to any cause before the completion of treatment); and other.

“Interruption of treatment during inpatient care” was defined as the occurrence of voluntary discharge or forced discharge from the study hospital, and “interruption of treatment during outpatient care” was defined as no reported tuberculosis treatment for at least 2 consecutive months during outpatient care before completion of treatment.

The social course identified in this study was independent of the definition of “tuberculosis treatment outcome” by the World Health Organization.^14^ In this study, while identifying interruption of treatment during inpatient or outpatient care, the subjects were followed-up even after interruption in treatment. Therefore, whether patients returned to treatment after an interruption for more than 2 months was confirmed.

### Data Analysis

The frequencies of demographic, social and clinical characteristics were calculated. The social course of the subjects based on the results of patient follow-up were plotted.

Statistical significance was examined by Fisher’s exact test for the following associations: the associations between demographic, social and clinical characteristics and interruption of treatment during inpatient care or death among all 119 patients; the associations between demographic, social and clinical characteristics and interruption of treatment during outpatient care among the 57 patients discharged from the study hospital for outpatient care. To estimate odds ratios of interruption of treatment during inpatient care and outpatient care or death, after adjustment for the influence of multiple variables that showed significant associations by univariate analysis, exact logistic regression analysis was performed. For these analyses, binary variables were used for residence before admission (homeless vs. non-homeless), residence after discharge (transient hostel vs. other residence), occupation (unemployed vs. employed), reason for access to medical services (found collapsed on the street vs. other reason), and result of sputum smear (‘-’ or 1+ or 2+ vs. 3+).

We compared the prevalence of drug-resistant *M. tuberculosis* isolates between the cases with and without a history of tuberculosis treatment.

The ethical appropriateness of the study protocol was reviewed and approved by the ethics committees of Tokyo Medical and Dental University and Tokyo National Hospital.

## RESULTS

### Demographic, Social, and Clinical Characteristics

Table 1 shows the demographic, social and clinical characteristics of the 119 subjects. The mean age of the subjects was 51.2 years (range, 24-73 years).

**Table 1.** Demographic, social, and clinical characteristics of the subjects.

Characteristic	n (%)
Sex	
Male	115 (97)
Female	4 (3)

Residence before admission*	
Homeless	50 (42)
Temporary residence	26 (22)
Residence near work place	19 (16)
Apartment	17 (14)
Other or unidentified residence	7 (6)

Residence after discharge	
Apartment	26 (22)
Social welfare institution^†^	19 (16)
Transient hostel	13 (11)
Apartment or house of family members	3 (3)
Temporary shelter	1 (1)
Hospital	27 (23)
Discharged dead or unknown ^‡^	30 (25)

Occupation before admission	
Unemployed	69 (58)
Construction worker and laborer	18 (15)
Service worker	9 (8)
Worker in transport	3 (3)
Sales worker	3 (3)
Other or unidentified occupation	8 (7)
Unknown	9 (8)

People with close relationships	
Any	32 (27)
None	82 (69)
Unknown	5 (4)

Reason for access to medical services	
Complaint of any symptom	79 (66)
Found collapsing on the street	19 (16)
Abnormal findings on health checks	15 (13)
Deterioration of tuberculosis during follow-up	5 (4)
Unknown	1 (1)

History of tuberculosis treatment	
Any	21 (18)
None	81 (68)
Unknown	17 (14)

Site of the disease	
Pulmonary tuberculosis	116 (98)
Extrapulmonary tuberculosis	3 (3)

Cavitary disease (n=116)	
Yes	94 (81)
No	20 (17)
Unknown	2 (2)

Sputum smear result^§^	
-	31 (26)
1+	16 (13)
2+	42 (35)
3+	27 (23)
Unknown	3 (3)

Drug resistance of M. tuberculosis isolates^∥^	
Yes	22 (18)
No	67 (56)
Unknown (culture positive)	15 (13)
Culture negative	15 (13)

Diabetes mellitus	
Yes	33 (28)
No	69 (58)
Unknown	17 (14)

Alcohol problems in the hospital	
Yes	9 (8)
No	94 (79)
Unknown	16 (13)

* : Residence before admission was categorized as follows: homeless (urban park, station building, riverbed, street, temporary winter accommodation for the homeless, or no residence); temporary residence (transient hostel, capsule or business hotel, public sauna, all-night theater, or apartment or house rented by friends); residence near work place (dormitory in workplace, or bunkhouse for construction workers); apartment (apartment rented by the patient and vacated because of disease onset); and others. Resident registration was assigned under the address of the hospital of the subjects’ admission, because of lack of a conventional dwelling or residence.

† : Social welfare institution includes hostel for the poor, public lodging for the homeless, rehabilitation facility, and rehabilitation institution for the internally handicapped.

‡ : Unable to obtain information on residence after discharge because of forcible or voluntary discharge.

§ : Results of sputum smear were classified as ‘-’, 1+, 2+, and 3+, equivalent to Gaffky scale of 0-1, 2-4, 5-8, and 9-10 respectively.

∥ : *M. tuberculosis* isolates were resistant to at least one of isoniazid, rifampin, ethambutol, or streptomycin.

### Social Course

**Figure 1.**
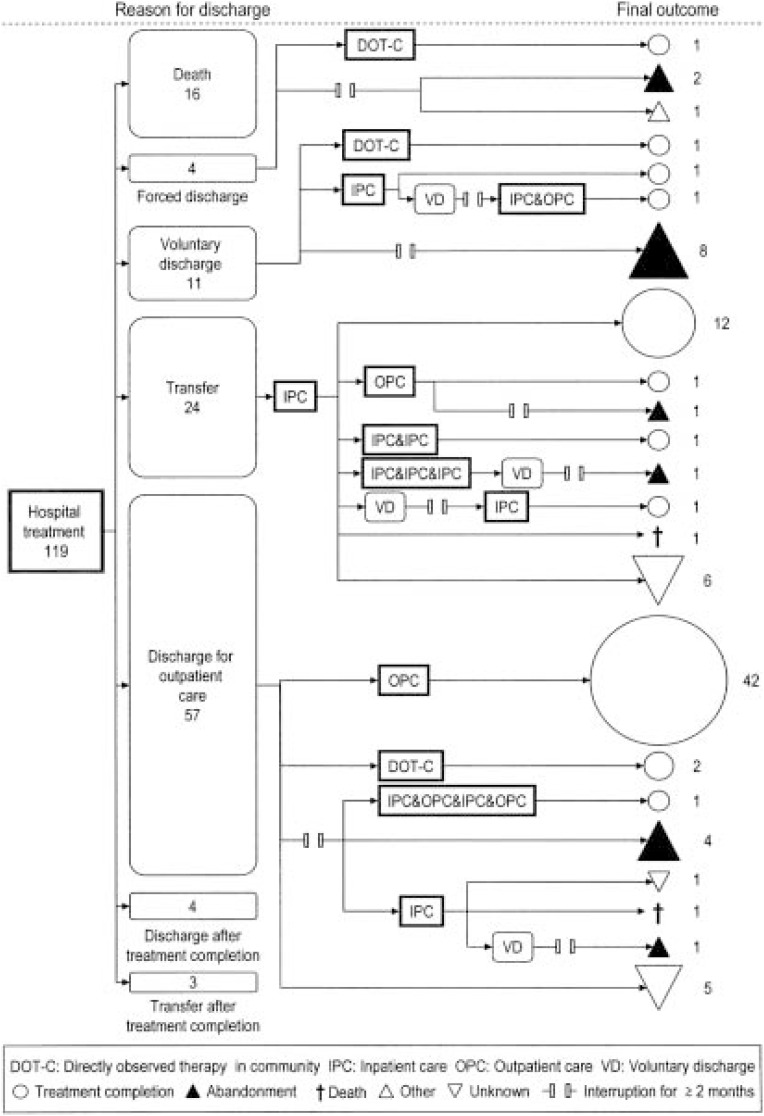
Social course of tuberculosis patients under fragile living conditions, from admission to hospital to final outcome. Reason for discharge (7 categories) is shown in boxes with rounded corners with the number of subjects in each category. Social course after discharge and final outcome (5 categories) are shown with arrows indicating individual or shared paths of the subjects. Types of medical services are indicated in block boxes with sharp corners.

Figure 1 shows the social course of the subjects. The total duration of follow-up from the day of admission through the final day to confirm treatment status was 2 to 1638 days (median, 342 days). Interruption of treatment during inpatient care at the study hospital occurred in 15 cases (4 cases of forced discharge and 11 of voluntary discharge). Interruption of treatment during outpatient care occurred in 3 out of 4 cases of forced discharge, 9 of 11 cases of voluntary discharge, 3 of 24 cases of transfer, and 8 of 57 cases of discharge for outpatient care. Eighteen (15.1%) patients died. After interruption for at least 2 months, treatment was resumed in 6 (26.1%) cases, and 3 (13.0%) of these cases completed treatment.

### Demographic, Social, and Clinical Characteristics and Interruption of Treatment and Death

Table 2 shows the percentage of cases of interruption of treatment and death according to demographic, social, and clinical characteristics. Table 2 also shows the results of exact logistic regression analysis by indicating adjusted odds ratios of selected characteristics. The percentage of subjects who interrupted treatment during inpatient care was higher among patients with alcohol problems in the hospital than in others (P<0.01). The proportion of cases with interruption of treatment during outpatient care were higher in patients who were homeless before admission and in those living in transient hostels after discharge than in others (P<0.01 and P<0.01). Adjusted odds ratio of living in a transient hostel after discharge showed an independent significant association with interruption of treatment during outpatient care. Mortality was higher in patients who were homeless before admission, in those found collapsed on the street than in others, and in those with sputum smear results of 3+ as compared with the others (P<0.01, P<0.001, and P<0.01). Adjusted odds ratios of those found collapsed on the street and sputum smear results of 3+ showed an independent significant association with the death of subjects.

**Table 2.** Interruption of treatment and death by demographic, social, and clinical characteristics.

Characteristic	Interruption of treatment						Death during inpatient or outpatient care		

	During inpatient care			During outpatient care					
		
	Total	n (%)	OR* (95% CI)	Total	n (%)	OR* (95% CI)	Total	n (%)	OR* (95% CI)
Sex									
Male	115	14 (12)		54	8 (15)		115	18 (16)	
Female	4	1 (25)		3	0 (0)		4	0 (0)	
		ns			ns			ns	

Age at admission (year)									
21-40	13	4 (31)		4	0 (0)		13	2 (15)	
41-60	90	10 (11)		48	8 (17)		90	11 (12)	
61-80	16	1 (6)		5	0 (0)		16	5 (31)	
		ns			ns			ns	

Residence before admission									
Homeless	50	9 (18)		15	6 (40)	8.0 (0.9, 116.4)	50	13 (26)	3.7 (0.7, 22.3)
Non-homeless	69	6 (9)		42	2 (5)	1.0	69	5 (7)	1.0
		ns			P<0.01	ns		P<0.01	ns

Residence after discharge									
Transient hostel	Not applicable			11	6 (55)	15.8 (1.8, 222.0)		‡	
Other residence				46	2 (4)	1.0			
					P<0.001	P<0.01			

Occupation before admission									
Unemployed	69	10 (14)		29	7 (24)		69	15 (22)	
Employed	41	4 (10)		25	1 (4)		41	3 (7)	
		ns			ns			ns	

People with close relationships									
Any	32	4 (13)		22	1 (5)		32	2 (6)	
None	82	11 (13)		33	7 (21)		82	16 (20)	
		ns			ns			ns	

Reason for access to medical services									
Found collapsing on the street	19	1 (5)		3	2 (67)		19	11 (58)	15.7 (3.3, 104.0)
Other reason	98	14 (14)		53	6 (11)		98	7 (7)	1.0
		ns			ns			P<0.001	P<0.001

History of tuberculosis treatment									
Any	21	5 (24)		10	2 (20)				
None	81	10 (12)		46	6 (13)				
		ns			ns				

Site of the disease									
Pulmonary tuberculosis	116	15 (13)		57	8 (14)		116	17 (15)	
Extrapulmonary tuberculosis	3	0 (0)		0	-		3	1 (33)	
		ns						ns	

Cavitary disease (among pulmonary tuberculosis cases)									
Yes	94	10 (11)		49	8 (16)		94	15 (16)	
No	20	3 (15)		8	0 (0)		20	1 (5)	
		ns			ns			ns	

Sputum smear result^†^									
‘ –’ or 1+ or 2+	27	2 (7)		11	0 (0)		89	9 (10)	1.0
3+	89	12 (13)		45	8 (18)		27	9 (33)	9.8 (1.9, 68.9)
		ns			ns			P<0.01	P<0.01

Drug resistance of *M. tuberculosis* isolates									
Yes	22	3 (14)		9	1 (11)		22	3 (14)	
No	67	6 (9)		40	6 (15)		67	7 (10)	
		ns			ns			ns	

Diabetes mellitus									
Yes	33	4 (12)		17	2 (12)			‡	
No	69	11 (16)		40	6 (15)				
		ns			ns				

Alcohol problems in the hospital									
Yes	9	5 (56)	10.1 (1.8, 60.1)	2	1 (50)			‡	
No	94	10 (11)	1.0	55	7 (13)				
		P<0.01	P<0.01		ns				

The association between demographic, social, and clinical characteristic and interruption of treatment during inpatient or outpatient care, and death were assessed by Fisher’s exact test.

* : Odds ratios were estimated by exact logistic regression analysis including characteristics with a P-value of less than 0.05.

† : Results of sputum smear were classified as ‘ - ’, 1+, 2+, and 3+, equivalent to Gaffky scale of 0-1, 2-4, 5-8, and 9-10, respectively.

‡ : Whether a characteristics is unknown or not is significantly associated with death.

CI: confidence interval

### Drug Susceptibility

The results of *M. tuberculosis* drug susceptibility tests are shown in Table 3. The prevalence of drug resistance was higher in cases with than without a history of tuberculosis treatment (P<0.05).

**Table 3.** Drug resistance of *Mycobacterium tuberculosis* isolates and history of tuberculosis treatment.

Drug resistance	History of tuberculosis treatment		

	Any	None	
	n = 15	n = 65	
	n (%)	n (%)	
Any of the four drugs	8 (53)	12 (19)	P<0.01
Isoniazid (0.1 *μ*g/mL)	5 (33)	3 (5)	P<0.01
Rifampin (10 *μ*g/mL)	6 (40)	1 (2)	P<0.01
Ethambutol (2.5 *μ*g/mL)	5 (33)	6 (9)	P<0.05
Streptomycin (20 *μ*g/mL)	5 (33)	6 (9)	P<0.05
Isoniazid and rifampin	3 (20)	0 (0)	P<0.01

Drug susceptibility of *M. tuberculosis* isolates was tested by the absolute concentration method. Critical concentrations were indicated in parentheses. Colony growth of at least 25% at the critical concentration was defined as indicating resistance.

The association between drug resistance of *M. tuberculosis* and a history of tuberculosis treatment was assessed by Fisher’s exact test.

## DISCUSSION

We determined the social course patterns of urban dwellers with tuberculosis lacking customary and regular access to conventional residences. Interruption of treatment during inpatient care was associated with alcohol problems, and that during outpatient care was associated with fragile living conditions. Patients under fragile-living conditions with a history of tuberculosis treatment showed a high prevalence of drug resistance. Patients who died before completion of tuberculosis treatment tended to have poor living conditions and were generally not admitted to hospital until the disease had progressed to an advanced stage.

The subjects of this study reasonably represented the follow-up data of tuberculosis patients lacking customary and regular access to a conventional dwelling or residence in the Tokyo area who had contacted medical services. The subjects followed-up were collected from the discharge records of the hospital with the largest number of tuberculosis beds in the Tokyo area. The demographic characteristics of the subjects and those of homeless populations in Tokyo in general and tuberculosis patients who had experienced homelessness in a national survey were similar in terms of age distribution and sex ratio.^3^^,^^10^^,^^11^^,^^15^^,^^16^ Further patients who stayed at the hospital for more than a year were less than 0.5% of all inpatients. Although the study population was not defined at diagnosis of tuberculosis and might be different in their characteristics when defined at diagnosis, therefore, the influences of this difference in selection methods on the characteristics of subjects and the study results were thought to be small. The standard tuberculosis treatment regimen applied to the subjects was in accordance with that recommended by the Ministry of Health.^17^ Completion of 89.9% follow-up aided in better understanding the social course patterns of urban dwellers with tuberculosis under fragile-living conditions.

Mortality in patients under fragile-living conditions in this study was 2.3 times higher than that in smear positive tuberculosis patients in general after adjustment for age and sex of the subjects in this study.^16^ There were two conditions meaningfully related to the death of patients. First, one in six subjects was taken to hospitals by ambulance after collapsing on the street; second, one fifth of the subjects showed large amounts of bacteria in their sputum. These findings suggest delay in the start of tuberculosis treatment in people under fragile-living conditions, and may relate to their high mortality. Health is not a priority of people under fragile-living conditions because of their immediacy of daily life,^18^ and such people generally do not access medical services until they have severe problems.^12^ Liaison with non-health sector workers should be encouraged to facilitate early detection of the disease and intervention to achieve appropriate treatment.

Many cases of interruption of treatment during inpatient care were reported, due to either forced or voluntarily discharge from the hospital before the completion of treatment. One-quarter of these cases showed positive sputum smears, with a potential to transmit tuberculosis within the community. Interruption of treatment during inpatient care was related to alcohol problems while in hospital. Alcohol-related health problems are generally common in people under fragile-living conditions.^3^ Alcohol abuse is associated with deficits in social skills, which is the ability to interact with people and basic skills to manage difficulties in daily life and to live productively.^19^ Thus, not only treatment of tuberculosis, but also care regarding the alcohol problems and programs to improve patients’ social skills should be provided simultaneously during treatment in such cases.

Interruption of tuberculosis treatment during outpatient care was closely related to fragile living conditions in people without access to a conventional dwelling or residence. The homeless and those using transient hostels were more likely to interrupt treatment during outpatient care. People who live in transient hostels often spend the daytime drinking alcohol on the street when they do not find employment.^20^ This lifestyle shows a lack of concern regarding their health, and results in difficulty in escaping the vicious circle of fragile living. Due to the high frequency of interruption of treatment, patients living in transient hostels during outpatient follow-up require special attention.

DOT programs in the community provide opportunities for those who have interrupted tuberculosis treatment to resume and complete the full treatment schedule. During the study period, DOT programs for patients under fragile-living conditions were provided in two areas in Tokyo: a metropolitan welfare center for day laborers and homeless people, and a public health center in the area with a homeless population of 1000.^21^ These DOT programs are now widely available at public health centers in communities. Effective measures to care for people under fragile-living conditions with DOT programs in the community must be shared with other communities to strengthen the capacity of public health services.

The prevalence of resistance to any of the four first-line drugs and that of multidrug-resistant tuberculosis were higher among the subjects with than in those without a history of tuberculosis treatment. Patients under fragile-living conditions were more likely to have a history of tuberculosis treatment as compared to the general population.^22^ Multidrug-resistant tuberculosis is associated with treatment failure and death, and is costly to treat.^23^^,^^24^ Therefore, increasing the effectiveness of treatment of patients under fragile-living conditions, by increasing adherence to the treatment regimen, is required to arrest the spread of drug-resistant tuberculosis.

The results of this study accounted for the accumulation of tuberculosis in this socially marginalized and disadvantaged population. Individuals affected by tuberculosis are likely to remain in obscure corners of the city until they become unable to survive in the urban environment, and thus there is not a significant chance of disease transmission within the community. A considerable number of patients under fragile-living conditions interrupt inpatient or outpatient care and disappear from the conventional social network without completion of tuberculosis treatment, although medical and welfare support systems provide these people with free access to treatment for tuberculosis and housing support. This results in recurrence of the disease and an increased prevalence of drug-resistance among the urban poor population. The already high prevalence of tuberculosis in this fragile population would increase further and might give rise to an epidemic if effective means to break the vicious circle are not implemented.

Examination of the social course of 119 urban dwellers with tuberculosis lacking customary and regular access to a conventional residence revealed a high likelihood of interruption of treatment in this population. Patients with alcohol problems showed an increased likelihood of discontinuation of hospital treatment as a result of forced or voluntary discharge. Those who were homeless and used transient hostels were more likely to interrupt outpatient care. These subjects were also suggested to lack access to medical care in the early stages of the disease. Delayed access to medical services and interruption of treatment constitute a vicious circle of tuberculosis in urban dwellers with poor living conditions. The care of patients with alcohol problems and programs to improve social skills should be integrated into inpatient treatment of tuberculosis patients under fragile-living conditions. Intensive follow-up of patients living in transient hostels during outpatient care would reduce the rate of treatment interruption by patients under fragile living conditions.

In conclusion, factors associated with interruption of tuberculosis treatment in patients under fragile-living conditions were identified. Interruption during inpatient care was significantly associated with alcohol problems and that during outpatient care was significantly associated with the use of transient hostels.

## References

[1] Barnes PF, Yang Z, Preston-Martin S, Pogoda JM, Jones BE, Otaya M, Patterns of tuberculosis transmission in Central Los Angeles. JAMA 1997; 278: 1159-63. 9326475

[2] Geng E, Kreiswirth B, Driver C, Li J, Burzynski J, DellaLatta P, Changes in the transmission of tuberculosis in New York City from 1990 to 1999. N Engl J Med 2002; 346: 1453-8. 12000815 10.1056/NEJMoa012972

[3] Takano T, Nakamura K, Takeuchi S, Watanabe M Disease patterns of the homeless in Tokyo. J Urban Health 1999; 76: 73-84. 10091192 10.1007/BF02344463 PMC3456708

[4] Gutierrez MC, Vincent V, Aubert D, Bizet J, Gaillot O, Lebrun L, Molecular fingerprinting of Mycobacterium tuberculosis and risk factors for tuberculosis transmission in Paris, France, and surrounding area. J Clin Microbiol 1998; 36: 486-92. 9466764 10.1128/jcm.36.2.486-492.1998 PMC104565

[5] Pablos-Mendez A, Knirsch CA, Barr RG, Lerner BH, Frieden TR Nonadherence in tuberculosis treatment: predictors and consequences in New York City. Am J Med 1997; 102: 164-70. 9217566 10.1016/s0002-9343(96)00402-0

[6] Center for Disease Control and Prevention Prevention and control of tuberculosis among homeless persons. Recommendation of the Advisory Council for the Elimination of Tuberculosis. MMWR 1992; 41: 13-22.

[7] Numata K Risk factor of nonadherence with tuberculosis therapy in Shinjuku-city’s tuberculosis patients and directly observed therapy services. Nippon Koshu Eisei Zasshi (Jpn J Public Health) 2002; 49: 58-63. (in Japanese) 11868345

[8] Toyota E, Ootani N, Matsuda Y, Tajima H An approach to the control of the so-called vagrant patients with tuberculosis. Kekkaku 1990; 65: 19-22. 2352407

[9] Nakanishi Y, Oyama Y, Takahashi M, Mori T A molecular epidemiological analysis of several outbreaks of tuberculosis in public saunas. A problem of tuberculosis among homeless people in the metropolitan area. Nippon Koshu Eisei Zasshi (Jpn J Public Health) 1997; 44: 769-78. (in Japanese) 9436385

[10] Kleinman A. The illness narratives: suffering, healing and the human condition. Basic Books. New York, 1988. 10.1097/ACM.0000000000001864 28952997

[11] Kleinman A. Social origins of distress and disease. Yale University Press, New Haven, 1986.

[12] National Hospital Organization Tokyo National Hospital. Pulmonary tuberculosis. [cited March 24, 2006]. Available from: http://www.hosp.go.jp/~tokyo/shinryou/kokyuki/tb.html. (in Japanese)

[13] Kido N Social work with tuberculosis patients with background of homelessness. Nippon Koshu Eisei Zasshi (Jpn J Public Health) 2000; 47: 894-9. (in Japanese) 11144159

[14] World Health Organization. Treatment of tuberculosis guidelines for national programmes, 3rd ed. World Health Organization, Geneva, 2003.

[15] Bureau of Welfare, Tokyo Metropolitan Government. Homeless people in Tokyo, for the establishment of a new system for welfare support. Bureau of Welfare, Tokyo Metropolitan Government, Tokyo, 2001. (in Japanese)

[16] Ministry of Health, Labour and Welfare. Report of emergent tuberculosis survey in 2000. Ministry of Health, Labour and Welfare, Tokyo, 2001. (in Japanese)

[17] Ministry of Health, Bureau of Health and Medicine, Division of AIDS, Tuberculosis and Infectious Disease. Standard of tuberculosis care and its manual. Japan Anti-Tuberculosis Association, Tokyo, 1996. (in Japanese)

[18] Levy BD, O’Connell JJ Health care for homeless persons. N Engl J Med 2004; 350: 2329-32. 15175433 10.1056/NEJMp038222

[19] Scaturo DJ, LeSure KB Symptomatic correlates of alcohol abuse as a presenting problem. J Clin Psychol 1985;41:118-23. 3973033 10.1002/1097-4679(198501)41:1<118::aid-jclp2270410121>3.0.co;2-4

[20] Discussion Council on Countermeasures for Sanya Problems. Future trend of countermeasures against Sanya problem. Bureau of Welfare, Tokyo Metropolitan Government, Tokyo, 2000. (in Japanese)

[21] Bureau of Hygiene, Tokyo Metropolitan Government. For the initiation of DOT program. Bureau of Hygiene, Tokyo Metropolitan Government, Tokyo, 2001. (in Japanese)

[22] Toyota E, Kobayashi N, Houjou M, Yoshizawa A, Kawana A, Kudo K Usefulness of directly observed therapy (DOT) during hospitalization as DOTS in Japanese style. Kekkaku 2003; 78: 581-5. (in Japanese) 14577344

[23] Espinal MA, Kim SJ, Suarez PG, Kam KM, Khomenko AG, Migliori GB, Standard short-course chemotherapy for drug-resistant tuberculosis: treatment outcomes in 6 countries. JAMA 2000; 283: 2537-45. 10815117 10.1001/jama.283.19.2537

[24] Mahmoudi A, Iseman MD Pitfalls in the care of patients with tuberculosis: common errors and their association with the acquisition of drug resistance. JAMA 1993; 270: 65-8. 8510299

